# Integrated Analysis of Genomic and Immunological Features in Lung Adenocarcinoma With Micropapillary Component

**DOI:** 10.3389/fonc.2021.652193

**Published:** 2021-06-17

**Authors:** Shirong Zhang, Yang Xu, Pan Zhao, Hua Bao, Xiyong Wang, Rui Liu, Rujun Xu, Jingjing Xiang, Hong Jiang, Junrong Yan, Xue Wu, Yang Shao, Jiafeng Liang, Qiong Wu, Zhihao Zhang, Shun Lu, Shenglin Ma

**Affiliations:** ^1^ Translational Medicine Research Center, Key Laboratory of Clinical Cancer Pharmacology and Toxicology Research of Zhejiang Province, Affiliated Hangzhou First People’s Hospital, Cancer Center, Zhejiang University School of Medicine, Hangzhou, China; ^2^ Research and Development, Nanjing Geneseeq Technology Inc., Nanjing, China; ^3^ Department of Pathology, Affiliated Hangzhou First People’s Hospital, Zhejiang University School of Medicine, Hangzhou, China; ^4^ Department of Thoracic Surgery, Hospital of Marine Police Corps, Jiaxing, China; ^5^ Department of Thoracic Surgery, Affiliated Hangzhou First People’s Hospital, Zhejiang University School of Medicine, Hangzhou, China; ^6^ School of Public Health, Nanjing Medical University, Nanjing, China; ^7^ Shanghai Lung Cancer Center, Shanghai Chest Hospital, Shanghai Jiao Tong University, Shanghai, China

**Keywords:** micropapillary adenocarcinoma, transcription termination factor 1, brain-specific angiogenesis inhibitor 3, tumor mutation burden, immunotherapy

## Abstract

**Background:**

Micropapillary adenocarcinoma is one of the most aggressive histologic subtypes of lung adenocarcinoma (LADC), and even a minor proportion of micropapillary component (MPC) within the LADC could contribute to poor prognosis. Comprehensive analysis of genetic and immunological features of LADC with different percentages of MPC would help better understand cancer biology of this LADC subtype and direct future treatments.

**Methods:**

We performed next-generation sequencing (NGS) for a discovery cohort of 43 LADC patients whose tumors were micro-dissected to separate MPC and non-MPC lesions and a reference cohort of 113 LADC patients. MPC-enriched genetic alterations that were detected in the discovery cohort were then confirmed using a validation cohort of 183 LADC patients. Immunological staining was also conducted on the MPC-containing samples in the discovery cohort.

**Results:**

Tumors with a higher percentage of MPC tended to harbor more tumor mutation burdens (TMBs) and chromosome instability (CIN). Some rare genetic events may serve as the genetic landscape to drive micropapillary tumor progression. Specifically, alterations in transcription termination factor 1 (*TTF1*), brain-specific angiogenesis inhibitor 3 (*BAI3*), mammalian target of rapamycin (*MTOR*), and cyclin-dependent kinase inhibitor 2A (*CDKN2A*) were cross-validated to be enriched in MPC-contained LADC. Additionally, tumors with a higher percentage of MPC were associated with a higher percentage of CD4+, CD8+, and PD-L1+ staining, and some genetic changes that were enriched in MPC, including *MET* amplification and *MTOR* mutation, were correlated with increased PD-L1 expression.

**Conclusion:**

We identified multiple novel MPC-enriched genetic changes that could help us understand the nature of this aggressive cancer subtype. High MPC tumors tended to have elevated levels of TMBs, T cell infiltration, and immunosuppression than low MPC tumors, implying the potential link between MPC content and sensitivity to immunotherapy.

## Introduction

Lung adenocarcinoma (LADC) is the most prevalent histologic type of non-small cell lung cancer (NSCLC), accounting for nearly 50% of all diagnosed cases worldwide (1). The World Health Organization classifies LADC into multiple histologic patterns, including lepidic, mucinous, acinar, papillary, solid, and micropapillary, with lung micropapillary adenocarcinoma being recognized as a poorly differentiated, high-grade tumor (2). Most LADCs are histologically heterogeneous and have a mixed growth pattern. The micropapillary component (MPC) in LADC has been considered as a poor prognostic marker, and even a small proportion of MPC in the primary tumor could contribute to disease progression and recurrence (2–4). Previous studies have revealed that tumors with MPC have a higher incidence of oncogenic mutations, such as *EGFR*, *KRAS*, and *BRAF*, compared with other histological subtypes (5, 6). The frequency of these oncogenic mutations was associated with the percentage of MPC in the entire tumor, although controversial results have been observed (7, 8). Nevertheless, the molecular features of LADC with MPC remain largely unknown.

The current treatment regimen for lung micropapillary adenocarcinoma includes surgical resection, chemotherapy, and targeted therapy. It has been shown that patients with the MPC suffered from increased local recurrence when treated with limited resection (9), whereas they might benefit from adjuvant chemotherapy during early tumor stages (10). As lung micropapillary adenocarcinoma patients have some targetable mutations, such as *EGFR* mutations, they could be treated with tyrosine kinase inhibitors (TKIs); however, almost all TKI therapies inevitably result in drug resistance. Recently, checkpoint inhibitor immunotherapy showed promising treatment effects against NSCLC. One of the critical factors that affect the efficacy of immunotherapy is the tumor microenvironment (11). Patients with certain immunological markers, such as higher tumor programmed death-ligand 1 (PD-L1) expression levels and T cell infiltrations, had promising responses to immune checkpoint inhibitor drugs (12, 13). Given the poor prognosis of patients diagnosed with MPC, assessing the tumor immunological microenvironment and determining whether patients with MPC could potentially benefit from these immunotherapies are of great clinical importance.

In this study, we used broad penal next-generation sequencing (NGS) technology to comprehensively analyze the genomic and immunological characterizations for MPC from LADC tumors using multiple LADC patient cohorts. We also assessed several immunological markers, including CD4, CD8, and PD-L1, in tumors with different percentages of MPC to evaluate the immunological microenvironment.

## Materials and Methods

### Patients and Samples

One hundred and fifty-six patients who were diagnosed with LADC and received surgery at Hangzhou First People’s Hospital were enrolled in this study. The surgically resected tumor samples were used to generate the formalin-fixed paraffin-embedded (FFPE) specimens, which were then proceeded for NGS and immunohistochemistry analyses. No patients received neoadjuvant therapy or targeted therapy before sample collections. This study was approved by the ethics committee of Hangzhou First People’s Hospital (ethical number: 2019-038-01). All patients signed informed consent forms for donating their samples to the tissue bank of Hangzhou First People’s Hospital and the study.

### Microdissection

Formalin-fixed paraffin-embedded (FFPE) specimens were reviewed by two pathologists independently to ensure tissue histological patterns and region of interest (ROI) for microdissection. Microdissection was conducted using AVENIO Millisect Dissection System (Roche Diagnostics Inc.). Specifically, the pathologists manually marked the ROIs with specific subtypes under microscopic guidance using the H&E reference slide and then save the marked picture as the reference image in the system. The slides waiting to be dissected were aligned to the marked reference image. The areas for dissection were transferred automatically from the H&E reference slide to the dissection slide, and the milling path was automatically generated. The tissue within ROI was dissected with milling tips and collected automatically in RNase-free tubes.

### Next-Generation Sequencing-Based Genomic Profiling

DNA was extracted from dissected FFPE tissue specimen using QIAamp DNA FFPE Tissue Kit (Qiagen). DNA concentration was measured with a Qubit 3.0 fluorometer (ThermoFisher, USA). DNA 1–2 μg was used for library construction using KAPA Hyper Prep kit (KAPA Biosystems). DNA Libraries were then used to generate target-enriched amplicons with Geneseeq Prime panel (425 cancer-related genes) (14). Constructed libraries were sequenced on Hiseq 4000 NGS platforms (Illumina). The experiment was performed in a centralized clinical testing center (Nanjing Geneseeq Technology Inc., China) following the protocol reviewed and approved by the ethical committee of Hangzhou First People’s Hospital.

NGS data were aligned to the hg19 reference human genome with the Burrows–Wheeler Aligner (bwa-mem) (15) and were then processed using the Picard suite (http://picard.sourceforge.net/) and the Genome Analysis Toolkit (GATK). MuTect was applied to paired normal and tumor BAM files for the identification of somatic single-nucleotide variants (16). Small insertions and deletions were detected using SCALPEL (http://scalpel.sourceforge.net). Tumor purity was estimated using ABSOLUTE (17). Purity-adjusted gene-level and segment-level copy number variations (CNVs) were calculated by CNV Kit (18). Chromosome instability score (CIS) was defined as the proportion of the genome with aberrant (purity-adjusted segment-level copy number ≥3 or ≤1) segmented copy number (19). Tumor mutation burden (TMB) was defined as the number of non-synonymous mutations per sample.

### Immunohistochemistry Staining and Analysis

Four-micrometer-thick tumor sections were stained for PD-L1 (clone 22C3, DAKO, Agilent, CA, USA), CD4 (clone BP6028; Biolynx, Hangzhou, China), CD8 (clone: EP334, Abcam, Cambrige, UK) using BenchMark automated immunostainer (Ventana, AZ, USA). Staining was evaluated in a blinded fashion by the pathologists. Scoring was assessed based on the proportion of positive cells among nucleated cells in the ROI. The positive cells were defined as tumor cells displaying membranous staining of PD-L1. The PD-L1 expression at MPC was quantified as the proportion of PD-L1-positive tumor cells in total tumor cells within the MPC area. The tumor proportion score (TPS) is a PD-L1 measurement that has been applied in clinical trials and in clinic of lung cancer (20). The percentage of CD4+ and CD8+ T cell in the peritumor region was assessed based on the proportion of positive T cells among nucleated cells in the peritumoral area, which was described in previous studies (21, 22).

### Statistical Analysis

Comparisons of proportion between groups were done using Fisher’s exact test. The trend of TMB and CIS was analyzed using two-sided Jonckheere’s trend test. Coupling the p values between the discovery cohort and the validation cohort was conducted using Fisher’s method. The unpaired two-sample Wilcoxon test was used for comparison between different groups. Two-sided p values of less than 0.05 was considered as statistically significant (*p < 0.05, **p < 0.01, ***p < 0.001, ****p < 0.0001). All statistical analyses were done in R (v.3.6.0).

## Results

### Patient Characteristics and Study Cohorts

We obtained surgically resected tumors and the matched white blood cell samples from 156 LADC patients who had no previous targeted treatment histories, including 43 patients whose tumors contained the MPC and 113 patients with LADC. The 43 patients, including one patient with five lung tumors and one patient with two lung tumors and one brain metastasis, were grouped as the discovery cohort (**Supplementary Table 1**). Of the 49 LADC tumor samples (from the 43 patients) within the discovery cohort, 21 tumors had a high percentage of MPC (30–100%), 27 tumors contained a low percentage of MPC (0 to 20%), and one tumor (from the patient with five lung tumors) does not have detectable MPC. The rest 113 LADC patients with other histologic subtypes were grouped as a reference cohort for further comparison with the discovery cohort (**Supplementary Table 2**). The validation cohort was obtained from a previously published dataset (23), including 17 MPC-predominant (MPP) samples and 166 non-MPP samples from European patients. As shown in **Table 1**, the demographic characteristics were generally similar in all three patient cohorts.

**Table 1:** The demographic characteristics of different patient cohorts.

Category	n (%)		
	Discovery cohort	Reference cohort	Validation cohort
	43 Asian MPC patients	113 reference Asian LADC patients	183 European LADC patients
**Median age, years (range)**	67 (39–79)	59 (27–80)	66 (36–87)
**Gender**			
Male	20 (46.5)	50 (44.2)	95 (51.9)
Female	23 (53.5)	56 (49.6)	88 (48.1)
NA	0 (0)	7 (6.2)	0 (0)
**Disease stage**			
I–III	39 (90.7)	89 (78.8)	148 (80.9)
IV	4 (9.3)	24 (21.2)	10 (5.5)
NA	0 (0)	0 (0)	25 (13.6)
**Smoking history**			
Yes	10 (23.3)	1 (0.9)	135 (73.8)
No	33 (76.7)	2 (1.8)	27 (14.8)
NA	0 (0)	110 (97.3)	21 (11.4)
**Ethnicity**			
Asian	43 (100)	113 (100)	10 (5.5)
European	0 (0)	0 (0)	169 (92.3)
Others	0 (0)	0 (0)	4 (2.2)

### Genetic Feature of Micropapillary Tumor Lesion in LADC

**Figure 1:**
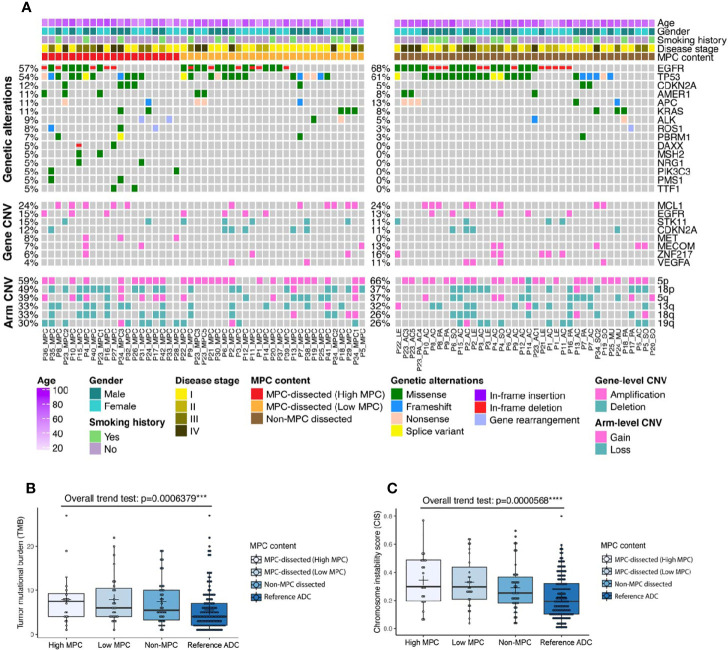
Genetic characteristics of micropapillary component (MPC)-containing tumors within the discovery cohort. **(A)** Genetic alteration profile of 43 MPC patients whose tumors were micro-dissected based on histologic subtypes and MPC content, including high MPC (30–100% of the original tumor), low MPC (0–20% of the original tumor), and the matched non-MPC regions. **(B, C)** The trend of tumor mutation burdens (TMB) **(B)** or chromosome instability score (CIS) **(C)** ranging from high MPC tumors, low MPC tumors, the matched non-MPC tumors to the reference LADC tumors.

After microdissection to separate different histologic subtypes of each tumor in the discovery cohort, we performed panel sequencing of 425 cancer-related genes for these samples. As one MPC-dissected sample did not pass the quality control, the sequencing data of 20 MPC-dissected samples from high MPC tumors, 27 MPC-dissected samples from low MPC tumors, and 38 matched non-MPC-dissected samples proceeded to subsequent analyses (**Supplementary Table 3**). When comparing all the MPC-dissected samples with non-MPC-dissected samples, the mutation profile was similar. There was no genetic alteration that was significantly enriched in a certain group (**Figure 1A** and Fisher’s exact test), although some unique alternations were found in MPC-dissected samples from the high MPC tumors, including cyclin-dependent kinase inhibitor 2A (*CDKN2A*) mutation, transcription termination factor 1 (*TTF1*) mutation, and mesenchymal–epithelial transition factor (*MET*) amplification (**Supplementary Table 4**). These data imply that the MPC and the matched non-MPC were likely to be derived from the same tumor-initiating cells and shared multiple genetic changes, and MPC tumors harbored some distinct alternations that might contribute to the formation of the MPC pattern.

We then performed the same panel sequencing for the samples from the reference cohort (**Supplementary Table 5**). As shown in **Supplementary Figure 1** and **Supplementary Table 6**, when comparing MPC-dissected samples from high MPC tumors of the discovery cohort with LADC samples of the reference cohort, some genetic alterations were significantly enriched in the high MPC group, including mutations of neurotrophic receptor tyrosine kinase 1 (*NTRK1*), myeloproliferative leukemia protein (*MPL*), malignant hyperthermia susceptibility 2 (*MHS2*), neurogenic locus notch homolog protein 2 (*NOTCH2*), *TTF1*, and APC membrane recruitment protein 1 (*AMER1*), amplification of *MET*, deletion of protein tyrosine phosphatase receptor type D (*PTPRD*), as well as some chromosomal arm-level changes, such as 18 loss, 6q loss, 11q, and 13q gain. Intriguingly, we were able to identify most of these genetic changes in all MPC-dissected samples, together with some additional genetic changes such as deletion of serine/threonine kinase 11 (*STK11*), *CDKN2A*, and *NOTCH1*, chromosomal arm 9q loss, and chromosomal arm 10p gain (**Supplementary Table 7**).

Based on the MPC percentage and the molecular similarity to the MPC, we ranked the samples from the two cohorts into the following order: MPC-dissected sample from high MPC tumors, MPC-dissected sample from low MPC tumors, the matched non-MPC-dissected samples, and the reference LADC samples. Strikingly, both tumor mutational burden (TMB) and CIS (chromosome instability score) tended to increase when moving towards higher MPC percentage/similarity (**Figures 1B, C**; Jonckheere’s trend test), indicating MPC-dissected samples, especially from high MPC tumors, were likely to have more mutation loads and chromosome instability. By analyzing the mutation frequency using the same ranked groups as above, we found that mutations in brain-specific angiogenesis inhibitor 3 (*BAI3*), AT-rich interactive domain-containing protein 2 (*ARID2*), cytochrome P450 2D6 (*CYP2D6*), and mammalian target of rapamycin (*MTOR*) had the trend to correlate with higher MPC percentage/similarity (**Supplementary Table 8**; Jonckheere’s trend test).

### MPC-Specific Molecular Features at Patient Levels

**Figure 2:**
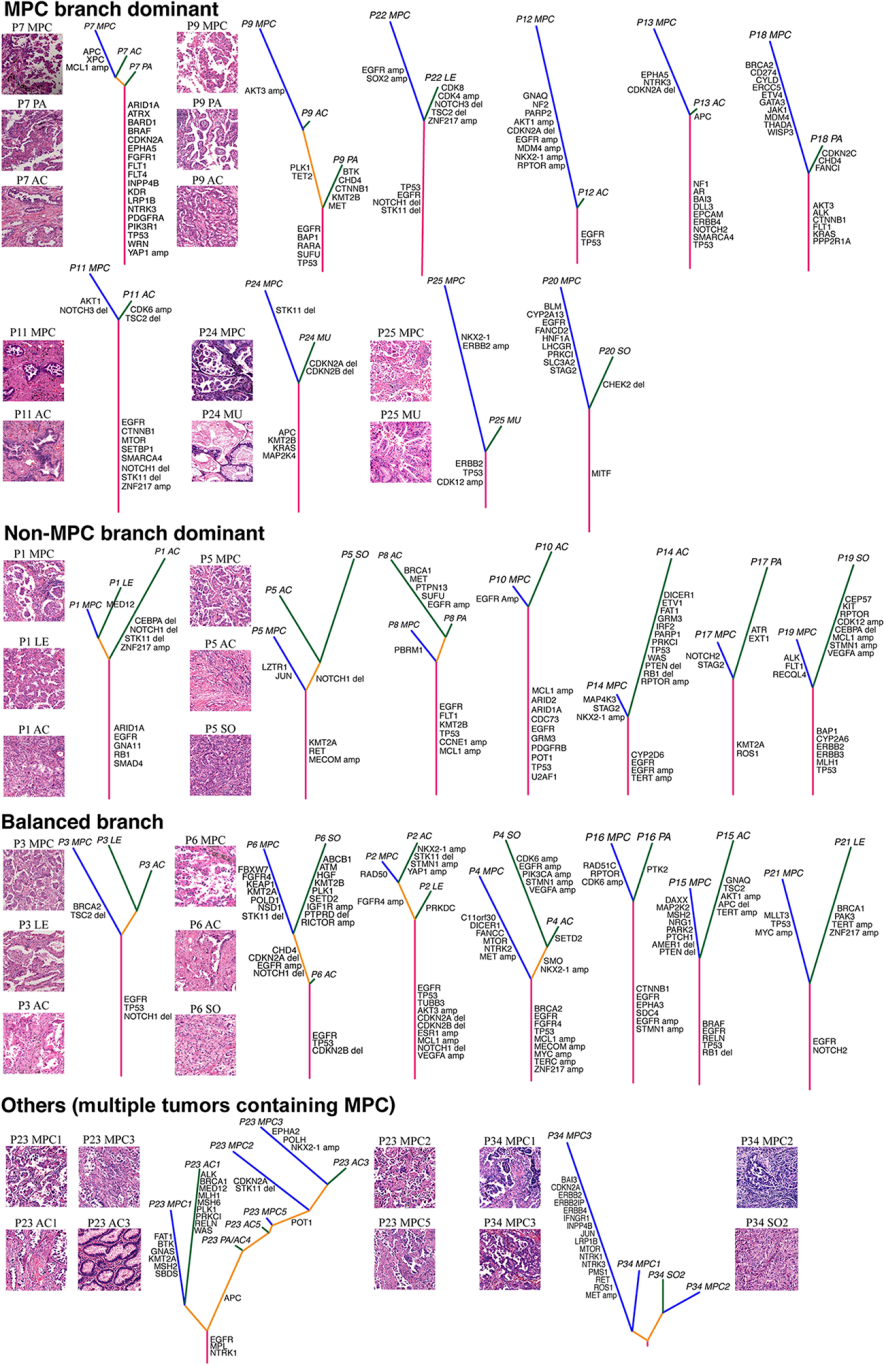
The molecular phylogenetic tree for each patient within the discovery cohort. The length of the stem and the branch represents the number of genetic alterations, including mutations, gene-level copy number variations (CNVs), and arm-level CNVs. Representative hematoxylin and eosin (H&E) staining of different histologic subtypes were illustrated. The mutations and gene-level CNVs were labelled on the branch of the phylogenetic tree. AC, acinar; LE, lepidic; MPC, micropapillary; MU, mucinous; PA, papillary; PA_AC, acinar and papillary mixture; SO, solid.

We then investigated the MPC-specific genetic changes at patient levels within the discovery cohort. As shown in **Figure 2**, the phylogenetic tree of different histologic subtypes was illustrated for each patient, and the length of trunks and branches was based on the number of gene mutations, gene-level CNVs, and arm-level CNVs. Representative hematoxylin and eosin (H&E) stains for each LADC subtype were shown for certain patients (**Figure 2**). Based on the branch length of MPC and non-MPC patterns, we separated patients who had only one original tumor into three groups: 1) MPC branch dominant group that has more genetic alterations in MPC-dissected sample; 2) non-MPC branch dominant group that has more genetic changes in the non-MPC-dissected sample; 3) balanced branch group that has similar branch lengths.

Generally, there were more patients in the MPC branch dominant group compared with those in the non-MPC branch dominant group (**Figure 2**), indicating that the MPC pattern might tend to accumulate more genetic alterations. In addition, the length of the trunk was significantly longer in the single original tumor groups than the multiple original tumor group (**Figure 2**). Of note, for each patient with multiple original tumors, the subtypes that were separated by microdissection tended to group together (**Figure 2**). Consistent with this observation, we found the different histologic patterns that were micro-dissected from the same tumor almost always had the same driver mutations (**Supplementary Figure 2A**), implying that mutations that confer subtype differences occurred after the tumor driver mutations. In contrast, only a few MPC-specific genetic alterations were found in each patient, such as stromal antigen 2 (*STAG2*) mutation, *MET* amplification, and *CDKN2A* deletion; however, none of them were significantly enriched (**Supplementary Figures 2B–D**; Fisher’s exact test). Therefore, it is relatively difficult to genetically separate different LADC subtypes of the same tumor as they tended to share multiple genetic events, especially for driver mutations.

### Validation of MPC-Enriched Genetic Alterations

**Figure 3:**
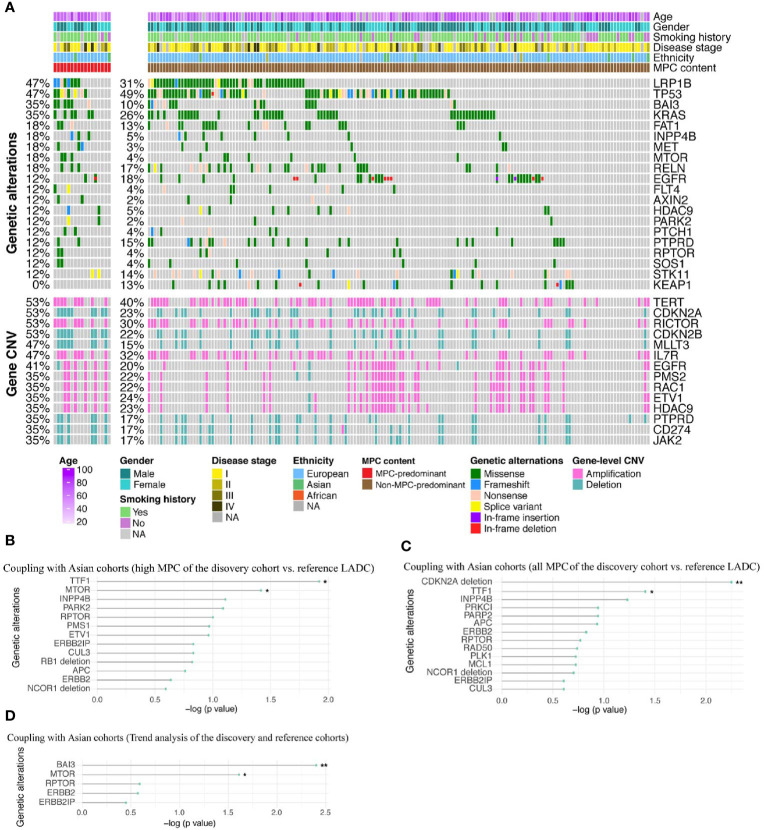
The MPC-associated genetic alterations that can be confirmed by the validation cohort. **(A)** Genetic alteration profile of the validation cohort with either MPC-predominant (MPP) tumors (left panel) or other histological subtypes (right panel). **(B–D)** The cross-validated MPC-associated genetic changes by coupling the P values from the validation cohort with the discovery/reference cohorts that underwent different analysis: comparing MPC-dissected samples from high MPC tumors of the discovery cohort with reference cohort **(B)**, comparing all MPC-dissected samples of the discovery cohort with reference cohort **(C)**, and Jonckheere’s trend analysis of mutation frequency ranking from dissected samples from high MPC tumors, dissected samples from low MPC tumors, the matched non-MPC dissected samples to reference LADC samples **(D)**. *p < 0.05, **p < 0.01.

Next, we used the validation cohort of 183 European LADC patients (23) to confirm the MPC-enriched genetic changes identified in the discovery cohort. The validation cohort was sequenced using whole-exome sequencing (WES) or whole-genome sequencing (WGS), so we only analyzed the genes that overlap with our panel sequencing to keep the consistency. As shown in **Figure 3A**, the top altered genes were LDL receptor-related protein 1B (*LRP1B*), *TP53*, *KRAS*, and telomerase reverse transcriptase (*TERT*), which are typically found in European LADC patients. Multiple genetic alterations were enriched in MPP samples compared with non-MPP samples, including *BAI3* mutation, *MET* mutation, *MTOR* mutation, *STAG2* amplification, p21 activated kinase 3 (*PAK3*) amplification, Bruton tyrosine kinase (*BTK*) amplification, SRY-box transcription factor 3 (*SOX3*) amplification, *CDKN2B* deletion, and *CDKN2A* deletion (**Figure 3A** and **Supplementary Table 9**). By coupling the p values of the discovery cohort and the validation cohort, several MPC-enriched genetic alterations could be cross-validated, including mutation of *TTF1*, *MTOR*, and *BAI3*, and deletion of *CDKN2A* (**Figures 3B–D**; Fisher’s method).

### Correlation Between the MPC Content and Immunological Markers

**Figure 4:**
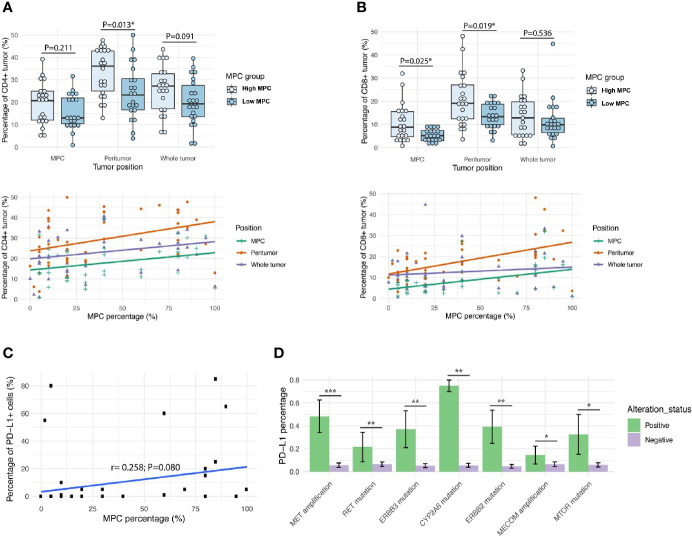
LADC tumors with higher micropapillary contents have more T cell infiltration and immunosuppression. The pattern of immune cell infiltrations of CD4+ **(A)** and CD8+ **(B)** detected in different regions of tumor and comparison of micropapillary tumor content and the immunological markers. Upper panel: high MPC tumors *vs*. low MPC tumors; lower panel: the MPC percentage of the whole tumor. The Pearson’s coefficient value (r) and the corresponding P value were attached for each immunostaining; **(C)** The scatterplot of MPC percentage of the original tumor and the percentage of PD-L1+ cells within the MPC; **(D)** Graphs showing the top genetic alterations that were associated with the percentage of PD-L1+ tumors. *p < 0.05, **p < 0.01, and ***p < 0.001.

Lastly, we investigated the immunological microenvironment of tumors with different percentages of MPC. We stained CD4 and CD8 for each tumor sample from the discovery cohort and analyzed the percentage of IHC positive cells of the whole tumor, the MPC region that was within the whole tumor, and the peritumor region that surrounded the whole tumor (**Figures 4A, B** and **Supplementary Figure 3**). Generally, the peritumor region and the whole tumor had higher CD4+ and CD8+ T cell percentage than the MPC region; on the other hand, compared with low MPC tumors, tumors with higher MPC tended to have more CD4+ and CD8+ T cell infiltration, especially at the peritumor regions (**Figures 4A–D**; **Supplementary Table 10**).

We also checked PD-L1 expression and found the percentage of PD-L1+ cells at the MPC region tended to be positively related to the MPC percentage of the original tumor (**Figure 4C** and **Supplementary Figure 4**). In addition, we found some genetic changes that were enriched in MPC, including *MET* amplification and *MTOR* mutation, were correlated with increased PD-L1 levels (**Figure 4D**), suggesting that these oncogenes might contribute to PD-L1 expression and immuno-suppression in MPC. Overall, our data suggest that the tumor with higher MPC tended to have more T cell infiltration and immunosuppression.

## Discussion

The implementation of precision medicine through molecular profiling has increasingly been integrated with standard clinicopathological evaluations to enhance diagnosis, prognostication, and personalized medicine for cancer patients (24). We reported here comprehensive genetic profiling for LADC with MPC. Our results revealed that MPC shared genetic abnormalities with other subtype components in the same tumor, indicating these tumor components rise from the same tumor-initiating cell. We also identified some genetic alternations that commonly occurred in the MPC, including genetic alternations in *NTRK1*, *MPL*, *MHS2*, *NOTCH1/2*, *TTF1*, *AMER1*, *STK11, MET*, *PTPRD, CDKN2A*, *BAI3*, and *MTOR*, and chromosome instability on chromosomal arms of 18q, 6p, 11q, 13q, 15q, and 9q. Of note, the clustering analysis showed these genes correlate to cancer metastasis (25), cancer cell reprogramming and cancer stemness (26–28), and/or cancer treatment resistance (29, 30). The characterization of these genetic alternations supports the clinical observations showing the association of the micropapillary pattern in LADC with the potential of metastasis/recurrence and poor diagnosis.

Mutations of *TTF1*, *MTOR*, and *BAI3* and deletions of *CDKN2A* were cross-validated to be enriched in the micropapillary tumor components using Asian and European patient cohorts. Interestingly, the *CDKN2A* gene encodes two proteins, p16^INK4a^ and p14^ARF^; p14^ARF^ can interact directly with TTF1 protein, and p14^ARF^ displays tumor suppressor activity in p53-negative cells partially through regulating ribosome biosynthesis and TTF1 localization (31). Therefore, the genetic alterations *of CDKN2A* and *TTF1* may contribute to the formation and/or maintenance of the micropapillary histology. On the other hand, we noticed the discrepancy of genetic alternations enriched in the micropapillary tumors between the Asian and European populations. For example, mutation of *BAI3* is found to be dramatically enriched in the micropapillary tumors of European patients, but not in Asian patients. *BAI3* is a member of the adhesion G protein-coupled receptor and it has been shown to be involved in diverse physiological and pathological conditions, including myoblast fusion, tumor progression, and neurological diseases (32). *BAI3* was found to be upregulated in small-cell lung cancer, although its function in these tumors was still unclear (33). Another example is *MET* amplification, which can be detected in high MPC tumors of Asian patients but not European patients. These findings indicate the potential impact of ethnicity on genetic drivers for developing micropapillary lung tumors.

Recently, immunotherapy has been introduced in clinical practice for the treatment of various types of cancer (34). T helper cell and cytotoxic T cell infiltration had positive prognostic significance in patients with NSCLC (35). In addition, large randomized clinical trials of targeting the programmed cell death protein 1 (PD-1) axis have demonstrated significant antitumor activities in some later-line metastatic diseases including lung cancer (36). In this study, we characterized immunological features of MPC, and we detected higher T cell infiltration and elevated expression of PD-L1 in high MPC tumors compared with low MPC tumors. These results indicate that micropapillary tumor cells might promote innate immune escape by forming an immunosuppressive microenvironment, and immunotherapy targeting PD-1/PD-L1 axis may thus benefit patients with micropapillary lung tumors.

There are several potential limitations in our study: 1) The discovery cohort only had 43 LADC patients, and it is difficult to identify MPC-specific genetic changes with such small sample size when the mutation frequency is relatively low; therefore, future studies with large patient cohort are necessary to further analyze the unique genetic features of MPC; 2) we did not perform microdissection for the reference cohort, which would reduce the sensitivity of identifying MPC-enriched mutations as tumor samples might be contaminated by a small percentage of MPC; 3) most of the patients in the discovery cohort were at very early disease stages and they did not have mature clinical results, so we lack the data to correlate the identified molecular/immunologic features with the clinical outcome; 4) there were only 2 patients with multiple original tumors in our discovery cohort, so we cannot compared the molecular features between the single original tumor and multiple original tumors; 5) as the discovery cohort were all Asian patients while the validation cohort was mainly comprised of European patients, our coupling analyses might miss some less frequent or race-specific molecular features.

## Conclusion

In summary, we demonstrated here that tumors with higher MPC harbor more TMBs and chromosome instability. We identified several micropapillary tumor-associated genetic alternations and hypothesized that these genetic events may drive intra-tumor heterogeneity and promote clonal/subclonal evolution of micropapillary pattern of LADC. Our data also revealed that the genetic events that were associated with the micropapillary tumors may also contribute to the development of an innate immune escape for tumor cells with inactivated PD-1/PD-L1 signaling, so immunotherapy strategy of combination anti-PD-1/PD-L1 with anti-CTLA-4 drugs may benefit patient with micropapillary lung cancer.

## Data Availability Statement

The data presented in the study were deposited in the Genome Sequence Archive for Human (GSA-Human) repository (https://bigd.big.ac.cn/gsa-human/), accession number (HRA000879).

## Ethics Statement

The studies involving human participants were reviewed and approved by the ethics committee of Hangzhou First People’s Hospital (ethical number: 2019-038-01). The patients/participants provided their written informed consent to participate in this study.

## Author Contributions

Conception and design: SZ, SM, and SL; administrative support: SZ, SM, and YS; provision of study materials or patients: XWa, HJ, and ZZ; collection and assembly of data: XWa, HJ, ZZ, PZ, JX, and RX; data analysis and interpretation: SZ, YX, JL, QW, HB, JY, RL, and XWu. Manuscript writing: all authors. All authors contributed to the article and approved the submitted version.

## Funding

This work was supported by grants from National Natural Science Foundation of China (81773242), Major project of Hangzhou Science and Technology Bureau (20180417A01), Projects of Science and Technology Project of Hangzhou Bureau (20170533B28, 20180533B98), and the Zhejiang Provincial Natural Science Foundation (LY19H160032).

## Conflict of Interest

YX, HB, RL, JY, XWu, and YS are employees of Nanjing Geneseeq Technology Inc.

The remaining authors declare that the research was conducted in the absence of any commercial or financial relationships that could be construed as a potential conflict of interest.

## Supplementary Material

Supplementary Figure 1 Comparison of the genetic alteration profiling between the MPC-high or MPC-low tumors of the discovery cohort (left panel) and the reference cohort (right panel).

Supplementary Figure 2 MPC specific genetic changes of micro-dissected tumors within the discovery cohort. **(A)** The driver mutations detected in each tumor with multiple histologic subtypes. **(B–D)** The frequency of MPC specific or non-MPC specific mutations **(B)**, gene-level CNVs **(C)**, and arm-level alterations **(D)** detected in each tumor with multiple histological subtypes.

Supplementary Figure 3 Representative immunohistochemistry (IHC) staining of tumor samples from the discovery cohort (patient P22). CD4 and CD8 staining in the micropapillary tumor component (left panels), the peritumor region (right panels, left portion from the red tumor boundary line), and the whole tumor (right panels, right portion from the red tumor boundary line).

Supplementary Figure 4 Representative IHC staining of tumor samples with PD-L1, CD4 and CD8 in low percentage of PD-L1+ cells (patient P3; PD-L1+ cells<1%), moderate percentage of PD-L1+ cells (patient P26; 1%≤PD-L1+ cells ≤ 50%), and high percentage of PD-L1+ cells (patient P10; PD-L1+ cells>50%).
