# Supplementary figures and images for: Integrated Analysis of Genomic and Immunological Features in Lung Adenocarcinoma With Micropapillary Component
Source: Front Oncol. 2021 Jun 17;11:652193. doi: 10.3389/fonc.2021.652193 (PMC8248503; doi:10.3389/fonc.2021.652193)

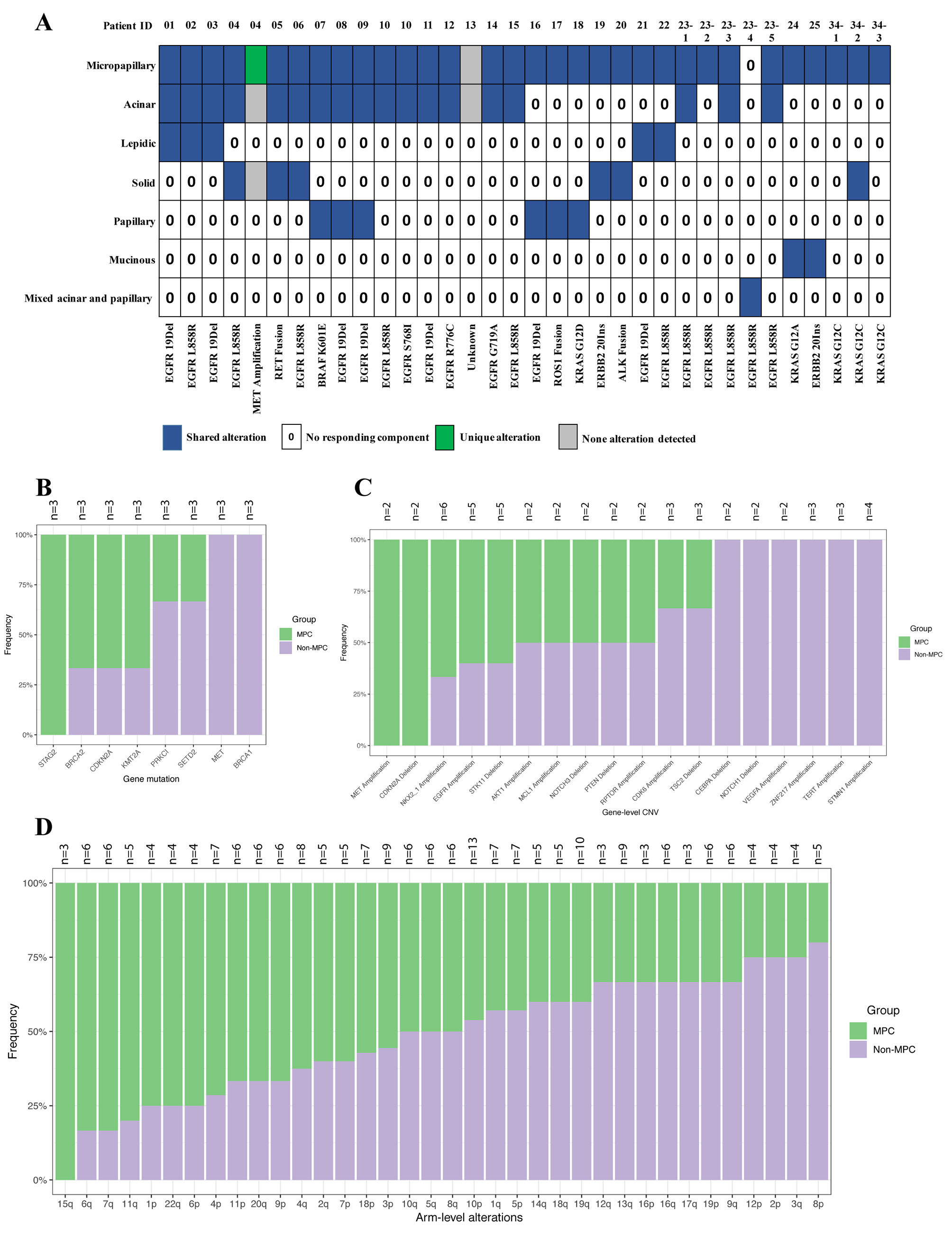

Supplement: Supplementary Figure 2 — MPC specific genetic changes of micro-dissected tumors within the discovery cohort. (A) The driver mutations detected in each tumor with multiple histologic subtypes. (B–D) The frequency of MPC specific or non-MPC specific mutations (B), gene-level CNVs (C), and arm-level alterations (D) detected in each tumor with multiple histological subtypes. [file Image_2.jpeg]

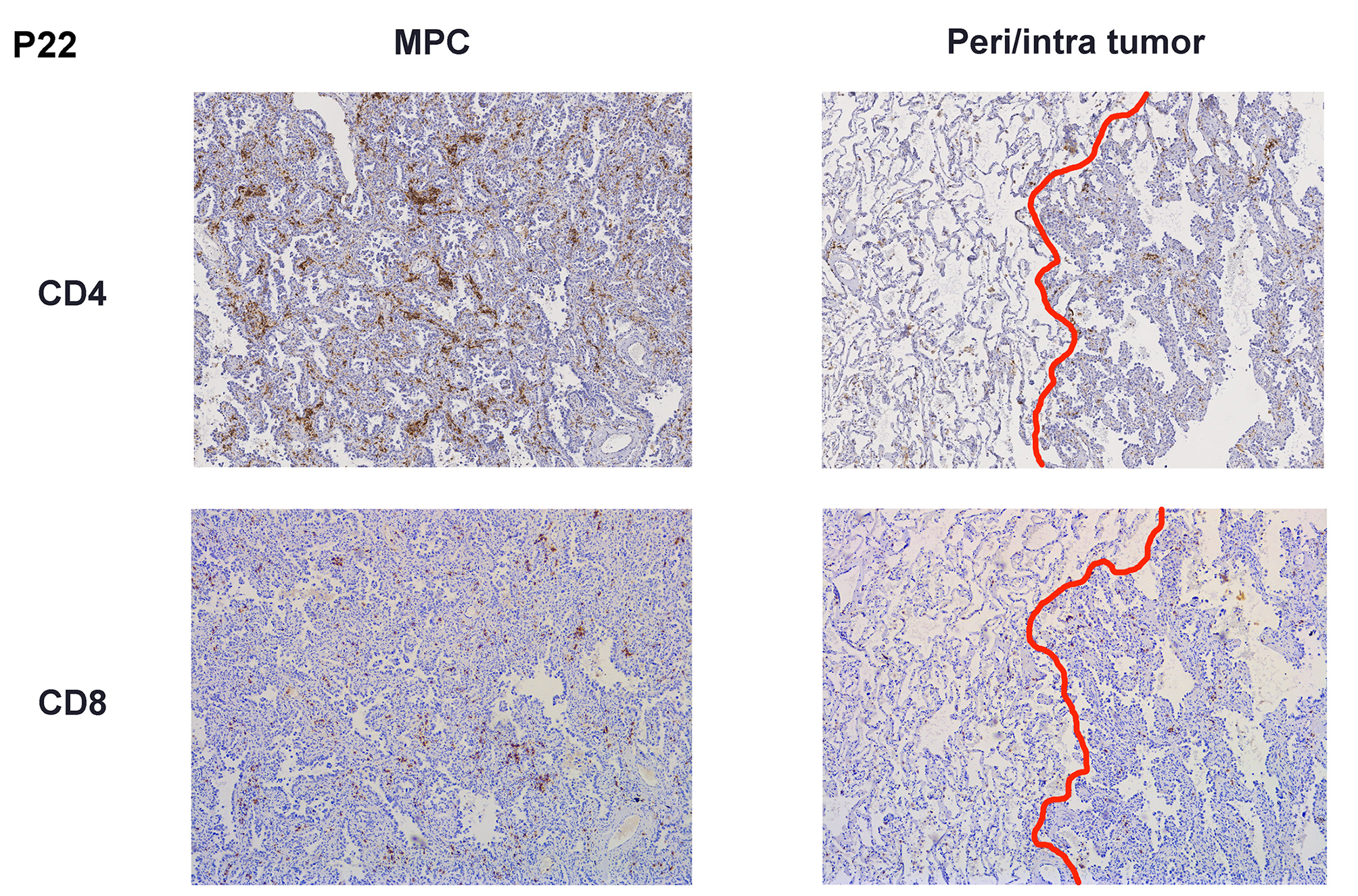

Supplement: Supplementary Figure 3 — Representative immunohistochemistry (IHC) staining of tumor samples from the discovery cohort (patient P22). CD4 and CD8 staining in the micropapillary tumor component (left panels), the peritumor region (right panels, left portion from the red tumor boundary line), and the whole tumor (right panels, right portion from the red tumor boundary line). [file Image_3.jpeg]
